# The Moderating Role of Empathy Profiles in the Relationship Between Knowledge About Aging and Attitudes Toward Older Adults Among Nursing Students

**DOI:** 10.3389/fpsyg.2021.713271

**Published:** 2021-10-18

**Authors:** Gui-Ying Yao, Yan-Yan Luo, Zhi-Min Zhao, Bo Zhu, Min Gao

**Affiliations:** ^1^School of Nursing, Xinxiang Medical University, Xinxiang, China; ^2^School of Nursing, Henan University Minsheng College, Kaifeng, China; ^3^Xiangya Nursing School, Central South University, Changsha, China

**Keywords:** empathy, knowledge about aging, nursing students, latent profile analysis, attitudes toward older people

## Abstract

Knowledge about aging (KA) and empathy affect nursing students’ attitudes toward older adults. However, little is known about the mechanisms underlying this phenomenon from an integrated, person-centered perspective. The purposes of the present study were (1) to identify empathy profiles based on the Interpersonal Reactivity Index (IRI) among Chinese nursing students and (2) to explore whether these latent empathy profiles moderate the association between KA and attitudes toward older people. A cross-sectional survey design was used, and a battery of questionnaires – including those on demographic information, the Chinese version of Palmore’s Facts on Aging Quiz (C-FAQ), the Chinese version of Kogan’s Attitude Toward Older People Scale (C-KAOP), and the IRI – was filled in by 622 Chinese nursing students (*M*_age_ 21.76; SD = 1.33). The mean total scores on KAOP and C-FAQ were 164.96 ± 18.32 and 10.436 ± 3.015, respectively, indicating relatively positive attitudes toward older people but low KA among Chinese nursing students. Latent profile analysis was used to identify a three-profile solution characterized by distinct levels of four dimensions of empathy, namely average empathy (AE, *n* = 399), high empathy (HE, *n* = 42), and low empathy (LE, *n* = 181). Subsequent linear regression analysis revealed that the LE rather than the HE profile predicted positive attitudes toward older adults. It is worth noting that the LE profile played a remarkable moderating role in associations between KA and negative attitudes toward older adults after controlling for covariant variables. Both the identification of distinct empathy profiles and the interplay between the LE profile and KA are of significance in reducing negative attitudes toward older adults among Chinese nursing students. Nursing educators should combine improving nursing students’ levels of KA and fostering greater empathy to reduce negative attitudes toward older adults. Such training should give priority to nursing students with LE.

## Introduction

The proportion of the global population that is elderly is increasing rapidly, especially in China, which accounts for one-fifth of the world’s population. In 2010, it was estimated that older people accounted for 8.2% of the population of China, and this figure is expected to increase to 23.9–26.9% of the total population by 2050 (Zeng, 2012). Demand for healthcare services is expected to grow accordingly, as older adults are at greater risk of suffering from multiple chronic diseases (Wang et al., 2020). The growing number of older adults means that more healthcare providers will be needed, among whom nurses make up the largest proportion. Nurses, including nursing students (who constitute a powerful reserve workforce), need to be equipped not only with professional knowledge about aging (KA) and skills but also with positive attitudes toward older adults. The latter is considered a key target for healthy aging (Rush et al., 2017).

According to the Age-Friendly Cities framework proposed by the World Health Organization [WHO] (2007), showing respect toward older patients is important in constructing age-friendly hospitals. Social exclusion among older adults and ageism toward them are found both in China and universally (Feng et al., 2019). Nurses with a positive attitude toward elderly people are better equipped to guarantee the provision of qualified care and to create age-friendly work environments (Kuo and Chen, 2019). Interventions to reduce ageism have been found to be most effective with younger adults and females (Burnes et al., 2019). Correspondingly, cultivating positive attitudes toward older adults in nursing students seems to be the best way to prepare for age-friendly caring in the future. Nursing students’ attitudes toward elderly people and related factors have thus been extensively studied under different cultures (Wanko Keutchafo et al., 2021). The findings have shown that the attitudes of nurses and nursing students toward older people are complex and subject to controversy (Rush et al., 2017), although findings have consistently shown that attitudes toward older adults are one of the best predictors of willingness to care for elderly people (Zhang et al., 2016; Abudu-Birresborn et al., 2019) and even have been found to influence the quality of healthcare services provided to older adults (Chai et al., 2019).

Many factors are involved in determining nursing students’ attitudes toward older adults, including demographic factors such as age (Salin et al., 2020), exposure to older adults (Liu et al., 2013), gerontological nursing education (Hsu et al., 2019), role modeling by clinical instructors (Gibbs and Kulig, 2017), and so on. Among these influencing factors, KA and empathy may be the most important (Abudu-Birresborn et al., 2019). Improving KA helped to foster a better understanding of older adults and to promote more positive attitudes toward older adults, ultimately contributing to the willingness of geriatric care (Chu and Chu, 2013).

Empathy has been defined as a multi-dimensional construct characterized by the ability to adopt others’ perspectives, respond emotionally to others’ negative experiences, and react in a sympathetic manner to others’ personal distress (PD), as well as the tendency to identify with emotions aroused by works of art (Davis, 1983). A large body of studies has focused on attitude improvement interventions that target empathy. For example, nursing educators have used simulation games such as the Senior Simulation Suit Programme (Cheng et al., 2020) and Geriatric Medication Game (Chen et al., 2015) to improve nursing students’ attitudes toward older adults by allowing them to experience the sensory and motor decline and functional impairment undergone during the aging process and thus trigger empathetic perceptions. Studies have also demonstrated that improved attitudes toward older people can be derived from activities such as simply wearing anything pertaining to being old, probably due to the increased empathy toward older adults this would generate (Cheng et al., 2020). An empathy-building task intervention was found to promote positive changes in the attitudes of medical students and doctors toward older people (Samra et al., 2013) and even to lead to improved care for elderly people in clinical nursing practice (Gholamzadeh et al., 2018).

Therefore, the roles of both KA and empathy as factors influencing attitudes toward older adults are well understood. However, it has been established that it is uncertain that interventions that seek to increase KA alone will change attitudes, indicating that just focusing on improving KA is not sufficient to change attitudes toward older adults (Samra et al., 2013; Cheng et al., 2020). Knowledge and attitudes have even been considered as distinct constructs (Samra et al., 2017). The reasons for the inconsistent associations between KA and attitudes toward aging have not been uncovered. Some other factors regarding attitudes toward older people, such as empathy, may be involved, given that interventions designed to increase KA with empathy components were found to change attitudes toward older adults effectively (Kaplan Serin and Tülüce, 2021). We thus hypothesized that empathy may modulate the association between KA and attitudes toward older adults.

Previous studies of the role of empathy in improving attitudes toward older adults have mainly discussed the issue from a variable-centered perspective (Kaplan Serin and Tülüce, 2021). However, empathy is heterogeneous and varies in accordance with gender, inherited traits such as ethnicity, and acquired traits such as college major (Davis, 1983; Zhao et al., 2019, 2020). Empathy can be explored through a person-centered lens to the benefit of targeted interventions (Wang et al., 2018; Laverdière et al., 2019). For example, a four-profile solution of empathy among psychotherapists and three different empathy profiles among preschool teachers have been identified (Wang et al., 2018; Laverdière et al., 2019). The nature of empathy profiles among nursing students is still elusive, despite the fact that the core value of empathy is stressed extensively in geriatric care (Teófilo et al., 2019).

Therefore, in the current study, our first step was to explore empathy profiles among nursing students from a person-centered perspective. As this is an exploratory analysis, no assigned profiles were hypothesized. Second, we hypothesized that the extracted empathy profiles may modulate the association between KA and attitudes toward older adults among nursing students. A better understanding of empathy profiles and the role of empathy among nursing students should be helpful in improving attitudes toward older adults, which is important in achieving age-friendly work environments (Kuo and Chen, 2019).

## Materials and Methods

### Participants

A cross-sectional study design was used, and 640 nursing students were recruited to participate in the current study between December 2019 and December 2020. The sample was sufficient according to the general criteria that the sample size should be at least 20 persons for each independent predictor in a linear regression model (Katz, 2003). The criteria for inclusion were that the respondents needed to be nursing students aged at least 18 years of age and to be registered as full-time undergraduates at two nursing schools in Henan province. A total of 622 of the eligible students returned valid questionnaires, and 18 were excluded because their responses were missing more than 20% of the required information.

The current study was approved by the Institutional Review Board and Ethics Committee of Xinxiang Medical University (XYLL-2019B007). Informed consent was obtained from the nursing students after explaining to them the study’s purpose and procedure. The participants were informed that they could enter and withdraw from the study freely, and the confidentiality of their information was maintained.

### Measures

The instruments used in the current study included a questionnaire on demographic characteristics such as age, gender, residence, only child or having siblings, and attitude-related questions such as whether they had taken geriatrics-related courses, whether they were raised by their grandparents in childhood, whether they had experience taking care of older adults, whether they were living with older adults such as grandparents, whether they had good relationships with older adults such as grandparents, and whether they were in contact with older adults such as grandparents.

The Chinese version of Palmore’s Facts on Aging Quiz (C-FAQ) was developed to examine physical, psychological, social, and economic KA and measure misconceptions regarding older adults (Wang et al., 2010). C-FAQ is a 25-item questionnaire with answers of “true” (T), “false” (F), or “don’t know.” A score of 1 is assigned if the answer is true. A higher score indicates a greater KA. The Cronbach’s α for C-FAQ in the present study was 0.862.

The Chinese version of the Kogan’s Attitude Toward Older People Scale (C-KAOP) was used to assess attitudes toward older people, including 17 negative (KAOP–) and 17 positive statements (KAOP+) (Yen et al., 2009). The KAOP– statements are reverse-scored, and a higher total KAOP score indicates a more positive attitude toward elderly people. A Likert scale 7-point system was applied (from strongly disagree to strongly agree with no score of 4). The Cronbach’s α in the current study was 0.797 for KAOP, and 0.873 and 0.848 for KAOP+ and KAOP–, respectively.

The Chinese version of the Interpersonal Reactivity Index (IRI) is a 5-point Likert self-rated questionnaire consisting of four subscales with 22 items, namely perspective-taking (PT), empathic concern (EC), personal distress (PD), and fantasy (FS). Cognitive empathy and emotional empathy were measured by PD and EC, respectively (Davis, 1983; Zhao et al., 2019). The Cronbach’s α for IRI in this study was 0.823.

### Statistical Analyses

Latent profile analysis (LPA) using the maximum likelihood estimator was used to identify the latent empathy profiles in Mplus 7.0. Specifically, to explore the optimal subgroups of empathy, smaller values of Akaike information criterion (AIC), Bayesian information criterion (BIC), and sample size-adjusted BIC (aBIC) were adopted to indicate better model fits. Moreover, entropy was used to evaluate the latent classification accuracy, following a previous study (Wang et al., 2017). Significant LMR and BLRT indicated that a k-profile model was superior to the *k*-1 one (Muthén and Muthén, 2012). Posterior classification probabilities were also used to further confirm the classification accuracy.

Descriptive statistics, including demographic characteristics, empathy, KA, and KAOP of Chinese nursing students, were performed in SPSS 24.0. A Chi-square test, *t*-test, one-way ANOVA, and follow-up *post hoc* tests were used to compare demographic characteristics within KAOP, KAOP+, and KAOP–. The standardized magnitude difference of empathy for each subgroup was determined by Cohen’s *d* effect sizes. According to Hidalgo and Goodman (2013), multiple linear regression analysis was used to explore the effect of empathy profiles on the relationship between KA and KAOP among nursing students. To clearly elaborate the roles of empathy profile in KAOP+ and KAOP– for designing tailored educational interventions, three regression models rather than a single regression model were used in this study.

## Results

### Descriptive Statistics of Study Variables Among Nursing Students in China

The means and standard deviations for KA, the different dimensions of empathy, and KAOP are presented in Table 1. The mean scores on KAOP and KA were 164.969 ± 18.321 and 10.436 ± 3.015, respectively, indicating relatively positive views of older adults but rather low knowledge of geriatrics among nursing students. The level of positive KAOP (85.359 ± 11.762) was higher than that of negative KAOP (79.610 ± 12.828). PT, EC, FS, and knowledge of geriatrics were significantly positively associated with KAOP. Age was only related significantly to KAOP– (*r* = 0.118, *p* = 0.003). As shown in Supplementary Table 1, nursing students whose characteristics were that they were living in an urban area (*p* = 0.005), were an only child (*p* = 0.004), had been raised by their grandparents in childhood (*p* = 0.014), had good relationships with their grandparents (*p* < 0.001 or *p* = 0.027) and maintained frequent contact with their grandparents (*p* < 0.001) exhibited higher levels of KAOP. Supplementary Tables 2, 3 show that KAOP+ and KAOP– were somewhat different among nursing students who were living in an urban area, had taken geriatrics-related courses, and had been raised by their grandparents in childhood, suggesting that demographic information was likely to impact KAOP+ and KAOP– among nursing students differently.

**TABLE 1:** Descriptive statistics and correlations of related variables used in the study (*n* = 622).

	Mean	SD	1	2	3	4	5	6	7	8
(1) KA	10.436	3.015	–							
(2) PT	13.015	2.698	0.153**	–						
(3) EC	15.272	2.895	0.092*	0.309**	–					
(4) PD	11.621	3.088	0.001	0.266**	0.082*	–				
(5) FS	15.294	3.159	0.115**	0.338**	0.433**	0.229**	–			
(6) KAOP	164.969	18.321	0.252**	0.209**	0.268**	–0.037	0.181**	–		
(7) KAOP+	85.359	11.762	0.211**	0.302**	0.243**	0.111**	0.214**	0.718**	–	
(8) KAOP^–^	79.610	12.828	0.167**	0.022	0.159**	–0.155**	0.062	0.770**	0.108**	–

KA, total scores of Palmore’s Facts on Aging Quiz; PT, perspective-taking; EC, empathic concern; PD, personal distress; FS, fantasy; KAOP, total scores of Kogan’s Attitude Toward Older People Scale; KAOP+, positive KAOP; KAOP−, negative KAOP. *Significant when *p* < 0.05. **Significant when *p* < 0.01.

### Identification of Empathy Profiles

**FIGURE 1:**
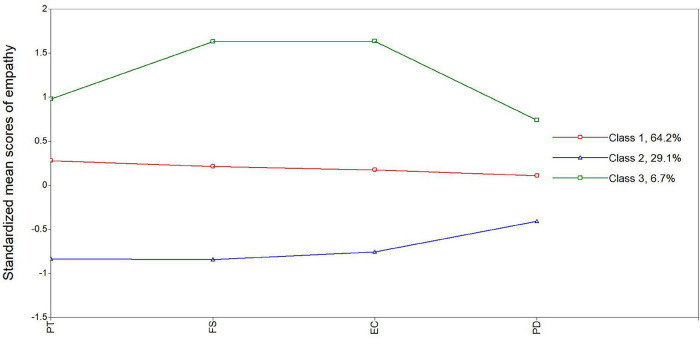
Plot of the standardized mean score of empathy across the three identified latent profiles among nursing students. PT, perspective-taking; EC, empathic concern; PD, personal distress; FS, fantasy.

With respect to the first main aim of our study, LPA was used to identify the latent empathy profiles among nursing students. Table 2 showed that LMR was significant for most profile solutions except a five-profile solution. The six-profile solution was excluded due to less than 1% of the sample size being in one class (Berlin et al., 2014). Although the entropy was only 0.709 in the three-profile solution, entropy was acceptable according to the prior study (Wang et al., 2017). As shown in Table 3, the posterior probabilities for each subgroup were 0.878, 0.854, and 0.856, respectively. Taken together, a three-profile was considered as the optimal solution and used for the following analyses. As shown in Figure 1. All three profiles were obviously distinguished according to scores of four dimensions of empathy. Specifically, the three-profile solution was labeled as high empathy (HE, 6.7%, *n* = 42), average empathy (AE, 64.2%, *n* = 399), and low empathy (LE, 29.1%, *n* = 181), respectively. The magnitudes of distinct groups were examined, and the results, which suggest a medium (Cohen’s *d* = 0.597) to large effect (Cohen’s *d* = 4.416) of magnitude difference across different empathy profiles, are shown in Table 4.

**TABLE 2:** Fit indices for six different latent empathy profiles (*n* = 622).

Fit indices	Number of profiles					
	1	2	3	4	5	6
AIC	7072.635	6878.484	6810.863	6753.933	6728.627	6703.195
BIC	7108.098	6936.113	6890.656	6855.890	6852.749	6849.482
aBIC	7082.700	6894.840	6833.509	6782.869	6763.854	6744.712
Entropy		0.540	0.709	0.704	0.699	0.742
LMR		0.004	0.011	0.012	0.068	0.013
BLRT		<0.001	<0.001	<0.001	<0.001	<0.001

*AIC, Akaike information criterion; BIC, Bayesian information criterion; aBIC, adjusted Bayesian information criterion; LMR, Lo-Mendell-Rubin; BLRT, bootstrapped likelihood ratio tests.*

**TABLE 3:** Average latent profile class probabilities for the most likely class membership (row) by latent class (column) (*n* = 622).

Latent class	The most likely class membership		
	AE	LE	HE
1	0.878	0.091	0.031
2	0.146	0.854	<0.001
3	0.144	<0.001	0.856

*AE, average empathy subgroup; LE, low empathy subgroup; HE, high empathy subgroup.*

**TABLE 4:** Mean, standard deviation of empathy and Cohen’s *d* of the three-profile solution of empathy among nursing students (*n* = 622).

	1AE (*n* = 399)		2LE (*n* = 181)		3HE (*n* = 42)				
	Mean	SD	Mean	SD	Mean	SD	*d*_2__–__1_	*d*_3__–__1_	*d*_3__–__2_
PT	13.83	2.14	10.34	1.90	16.03	2.56	1.725	0.932	2.524
PD	11.93	3.03	10.26	2.54	14.52	3.30	0.597	0.817	1.447
FS	16.05	2.40	12.22	2.05	21.12	1.98	1.717	2.304	4.416
EC	15.82	2.41	12.83	1.92	20.52	1.87	1.372	2.179	4.058

*PT, perspective-taking; PD, personal distress; FS, fantasy; EC, empathic concern; AE, average empathy subgroup; LE, low empathy subgroup; HE, high empathy subgroup.*

### Associations of Knowledge, Empathy Profiles, and Attitudes toward Older Adults Among Nursing Students

Regarding the second aim of the current study, multiple linear regression was used to examine the direct and interactive effects of KA and different empathy profiles on KAOP, taking demographic variables into account. AE was regarded as the reference for comparing with LE and HE. As shown in Table 5, *R*^2^ = 0.189, *F*(10,611) = 14.234, and *p* < 0.001, thus, the model for KAOP could explain 18.9% of the variance. According to magnitude of standardized *β* coefficient, knowledge about aging (*β* = 0.279), having a good relationship with older adults such as grandparents (*β* = −0.168), and different levels of empathy (*β* = −0.184, 0.092 for LE and HE compared with average level of empathy, respectively) significantly predicted KAOP. Knowledge about aging seemed more important (*β* = 0.279) than other predictors in the model to KAOP. Moreover, nursing students who fell within the HE profile were more likely than their counterparts who fell within the AE profile to show higher levels of KAOP. It is worth noting that KA and the LE profile had significant interactions in negatively predicting KAOP (*β* = −0.139, *p* = 0.003). However, no interaction was found between KA and the HE profile. These results indicated that students with LE may hold negative attitudes about older adults despite having sufficient knowledge about aging.

**TABLE 5:** Regression analysis predicting KAOP from KA and empathy profiles.

Variables	*B*	SE	*β*	95% CI		*t*	*p*
Residence	2.112	1.606	0.052	–1.042	5.266	1.315	0.189
Only child	–3.945	2.005	–0.078	–7.882	–0.008	–1.968	0.050
**Raised by grandparents**							
	–2.775	1.566	–0.066	–5.851	0.031	–1.771	0.077
**Having good relationships with older adults such as grandparents**							
	–7.252	1.861	–0.168	–10.906	–3.598	–3.898	<0.001
**Contact with older adults such as grandparents**							
	–1.974	1.094	–0.078	–4.121	0.174	–1.805	0.072
KA	1.694	0.291	0.279	1.123	2.265	5.829	<0.001
**Empathy profiles**							
LE vs. AE	–7.609	1.560	–0.184	–10.673	–4.546	–4.878	<0.001
HE vs. AE	7.497	3.027	0.092	1.553	13.441	2.477	0.014
**KA × empathy profiles**							
KA × LE	–1.413	0.477	–0.139	–2.351	–0.476	–2.966	0.003
KA × HE	–0.898	1.138	–0.030	–3.134	1.338	–0.789	0.431
Unadjusted *R*^2^ 0.189							

*KA, total scores of Palmore’s Facts on Aging Quiz; KAOP, total scores of Kogan’s Attitude Toward Older People Scale; AE, average empathy subgroup; LE, low empathy subgroup; HE, high empathy subgroup.*

To be more specific, Table 6 shows that nursing students who have siblings (*β* = −0.077), had a good relationship with older adults (*β* = −0.170), and maintained adequate KA (*β* = 0.202) and fell within the LE profile (*β* = −0.257) rather than the HE profile predicted 16.9% (*R*^2^ = 0.169, *F* (10,611) = 12.383, *p* < 0.001) of the variance of KAOP+ among nursing students, suggesting that LE (*β* = −0.257) was more important factor for positive attitude toward older adults. No interactions between KA and HE or LE were found, suggesting that the association of knowledge about aging and positive attitude toward older people was not regulated by different empathy profiles.

**TABLE 6:** Regression analysis predicting KAOP+ from KA and empathy profiles.

Variables	*B*	SE	*β*	95% CI		*t*	*p*
Only child	–2.504	1.208	–0.077	–4.877	–0.131	–2.072	0.039
**Having taken geriatrics-related courses**							
	–0.207	0.905	–0.009	–1.983	1.569	–0.229	0.819
**Raised by grandparents**							
	–1.997	1.017	–0.074	–3.994	0.000	–1.964	0.050
**Having good relationships with older adults such as grandparents**							
	–4.718	1.216	–0.170	–7.106	–2.330	–3.880	<0.001
**Contact with older adults such as grandparents**							
	–0.180	0.709	–0.011	–1.572	1.213	–0.254	0.800
KA	0.788	0.189	0.202	0.417	1.158	4.175	<0.001
**Empathy profiles**							
LE vs. AE	–6.813	1.030	–0.257	–8.835	–4.791	–6.618	<0.001
HE vs. AE	3.756	1.979	0.072	–0.131	7.643	1.897	0.058
**KA × empathy profiles**							
KA × LE	–0.595	0.310	–0.091	–1.204	0.014	–1.918	0.056
KA × HE	–0.236	0.740	–0.012	–1.690	1.218	–0.319	0.750
Unadjusted *R*^2^ 0.169							

*KA, total scores of Palmore’s Facts on Aging Quiz; KAOP, total scores of Kogan’s Attitude Toward Older People Scale; KAOP+, positive KAOP; AE, average empathy subgroup; LE, low empathy subgroup; HE, high empathy subgroup.*

**FIGURE 2:**
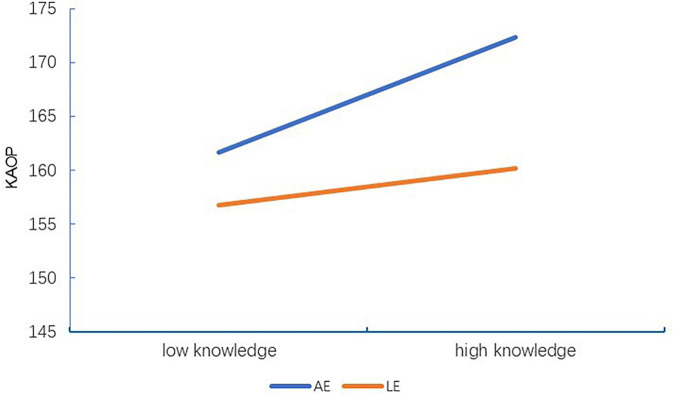
Interaction effect of empathy profile and knowledge of aging on attitudes toward older adults among nursing students. KAOP, total mean scores of Kogan’s Attitude Toward Older People Scale; AE, average empathy subgroup; LE, low empathy subgroup.

**FIGURE 3:**
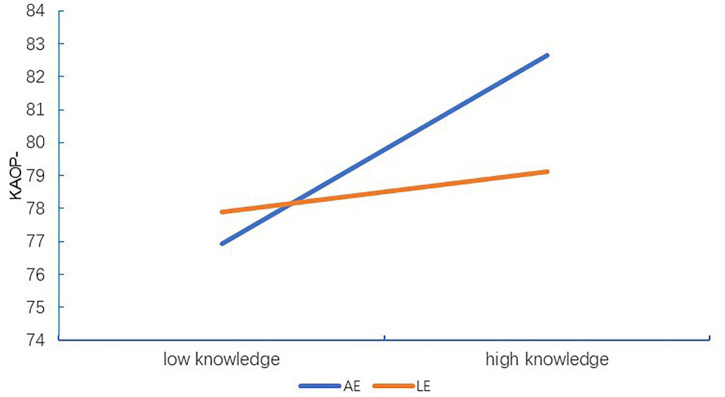
Interaction effect of empathy profile and knowledge of aging on negative attitude toward older adults among nursing students. KAOP, total mean scores of Kogan’s Attitude Toward Older People Scale; KAOP–, negative KAOP; AE, average empathy subgroup; LE, low empathy subgroup.

In terms of *R*^2^ = 0.077, *F*(9,612) = 5.655, and *p* < 0.001 shown in Table 7, only 7.7% of the variance of KAOP– was predicted by less frequent contact with older adults (*β* = −0.108) and KA (*β* = 0.213) and interactions between KA and the LE profile (*β* = −0.115). However, empathy profiles were not involved in predicting KAOP– on their own, suggesting that the LE profile merely acted as a modulator regulating the effects of KA on KAOP– (*β* = −0.115, *p* = 0.021). As shown in Figures 2, 3, the buffering effects of the LE profile on the relationships between KA and KAOP or KAOP– suggest that for nursing students who fall within the LE profile, attitudes toward older adults improve slowly as KA increases, compared to the larger magnitude of change in this regard seen among their counterparts in the AE profile.

**TABLE 7:** Regression analysis predicting KAOP– from KA and empathy profiles.

Variables	*B*	SE	*β*	95% CI		*t*	*p*
Residence	1.237	1.197	0.044	–1.113	3.588	1.034	0.302
Only child	–1.888	1.496	–0.053	–4.825	1.049	–1.262	0.207
**Having good relationships with older adults such as grandparents**							
	–2.582	1.386	–0.085	–5.303	0.139	–1.864	0.063
**Being in contact with older adults such as grandparents**							
	–1.909	0.808	–0.108	–3.496	–0.323	–2.363	0.018
KA	0.906	0.216	0.213	0.481	1.331	4.183	<0.001
**Empathy profiles**							
LE vs. AE	–0.736	1.164	–0.025	–3.021	1.549	–0.632	0.527
HE vs. AE	3.683	2.258	0.064	–0.751	8.118	1.631	0.103
**KA × empathy profiles**							
KA × LE	–0.821	0.356	–0.115	–1.520	–0.122	–2.306	0.021
KA × HE	–0.666	0.849	–0.032	–2.332	1.001	–0.784	0.433
Unadjusted *R*^2^ 0.077							

*KA, total scores of Palmore’s Facts on Aging Quiz; KAOP, total scores of Kogan’s Attitude Toward Older People Scale; KAOP–, negative KAOP; AE, average empathy subgroup; LE, low empathy subgroup; HE, high empathy subgroup.*

## Discussion

The present study aimed to identify the latent profiles of empathy based on IRI among nursing students and to examine the extent to which empathy profiles moderate the relationship between knowledge about aging and attitude toward older adults. Our study enriches previous findings on nursing students’ empathy from a person-centered perspective by identifying three distinct profiles labeled as HE, AE, and LE subgroups among nursing students. Furthermore, the LE profile was found to act as a moderator modulating the magnitude of the association between knowledge of geriatrics and attitudes toward older adults. The results highlight the fact that it is not sufficient for nursing students to be merely empowered with knowledge of geriatrics to reduce negative attitudes toward older adults. Nursing educators should cultivate nursing students’ capacity for empathy with a view to fostering an age-friendly healthcare environment and high-quality nursing practice in China’s forthcoming aging society (Kuo and Chen, 2019).

First, majority of our findings, such as lower KA were found to be consistent with previous studies (Liu et al., 2013; Samra et al., 2017; Hsu et al., 2019), indicating that a lack of adequate knowledge about aging is a pervasive concern across cultures. Thus, interventions and educational programs designed to enhance knowledge of geriatrics and to improve KAOP are generally required and of referential significance, given that geriatric knowledge plays a causal role in determining attitudes toward older adults according to attitude–behavior consistency theory (Fabrigar et al., 2006).

Improving knowledge about aging is considered fundamental in promoting positive KAOP (Donizzetti, 2019). Besides, nursing students’ empathy is also a key component for KAOP and high-quality care for elderly people (Kaplan Serin and Tülüce, 2021). Merely enhancing knowledge does not consequently change KAOP, but interventions involving empathy or simply focusing on empathy-building strategies do successfully improve attitudes toward older adults (Samra et al., 2013; Gholamzadeh et al., 2018; Cheng et al., 2020), indicating that empathy may play an important but underestimated role.

We thus explored the mechanisms from a person-centered perspective. First, consistent with a previous study among preschool teachers based on affective and cognitive empathy (Hojat and Gonnella, 2017), a three-profile solution of empathy was also found in our study. The proportion of the AE (64.2%) group, characterized by slightly above-average levels of empathy, was much larger than the moderate subgroup among preschool teachers (25.4%) (Wang et al., 2018) or the AE subgroup among psychologists (38%) (Laverdière et al., 2019), suggesting an adequate and even distribution of every dimension of empathy among nursing students. That majority of nursing students fell within the AE profile was also consistent with the findings of a previous study from a variable-centered perspective which found that most nursing students’ empathic tendency was above the moderate level in Turkey (Sağır and Bal Özkaptan, 2016). Similarities regarding empathy classification among nursing students from both variable- and person-centered approaches strongly support the efficacy of the current study’s classification scheme.

Nearly one-third of nursing students (29.1%) exhibited the lowest levels of empathy (labeled as LE), strongly suggesting that special importance should be attached to empathy training both in school education and continued education in view of the trends for empathy to decline over the period of clinical practice (Ten Hoeve et al., 2017; Chai et al., 2019). For example, interventions with immersive and experimental simulations concentrating on older adults could be applied to improve empathy (Levett-Jones et al., 2019).

A total of 6.7% of nursing students were found to be characterized by the highest levels of all four facets of empathy. Nursing students in this subgroup not only exhibited a good understanding of older adults’ feelings and shared emotional responses with elderly people but were also at high risk of becoming overly self-absorbed due to experiencing the highest level of emotional distress. Excessively self-involved empathy toward older adults may be detrimental in terms of creating a vulnerability to compassion fatigue and burnout (Duarte et al., 2016).

Although higher empathy was closely linked to attitudes toward the acceptability of aging (Waldrop et al., 2016) and was essential to improve the positive perceptions of old age (Sağır and Bal Özkaptan, 2016), we determined that the effect of KA on KAOP was only weakened in the LE rather than the HE subgroup among nursing students. Nursing students with relatively higher empathy may be more positive and more altruistic to older adults (McCamant, 2006). However, extremely high affective empathy and PD are likely to lead to emotional vulnerability and increase the risk factor for compassion fatigue (Duarte et al., 2016). More vicarious emotional distress was found in nursing students in practice care (López-Pérez et al., 2013). Attention should also be paid to nursing students with HE. The result that LE plays a remarkable role in modulating the magnitude of associations between KA and KAOP– has important educational implications. More interventions to reduce negative attitudes toward older adults rather than to promote more positive attitudes are needed to target knowledge about aging and LE synchronously.

Inconsistently with previous studies (Waldrop et al., 2016), we found that having good relationships but not increasing contact with older adults contributed to KAOP+. On the one hand, respect and reciprocity relationships contribute greatly to KAOP+ among healthcare students (Marchetti et al., 2021). On the other hand, frequent close contact with older adults has mainly been explored in clinical settings, and attitudes toward older patients are slightly different from those toward older adults in general (Costa et al., 2017).

Only18.9, 16.9, or even 7.7% variance of KAOP, KAOP+, and KAOP– could be explained in the current regression models. Consistent with previous studies, those results reflected the fact that attitude toward older people might be influenced by many factors such as environment including cultural settings and societal values, education, experience, and demographics (Rush et al., 2017; Abudu-Birresborn et al., 2019), indicating multiple efforts should be taken to promote positive attitude toward older people together with knowledge providing and empathy trainings.

It should be noted that the current study has some limitations. First, as the participants of the study were nursing students from two universities in Henan province where filial piety is strongly advocated in Chinese Central plain culture, the generalizability of the results to other cultural settings may be limited. Future studies are needed to examine the cross-cultural characteristics of the empathy profiles and their moderating roles. Second, longitudinal rather than cross-sectional designs could facilitate clarification of the nature and roles of empathy profiles, given the trend toward declining empathy among nursing students (Andersen et al., 2020). Third, convenience sampling may have led to bias that could be addressed by adopting nationally representative samples in future studies. Moreover, just as a common concern associated with the application of multivariable analysis, multiple linear regression could only eliminate some confounding (Katz, 2003). The current results only adjusted for some variables measured in this study, other unmeasured factors might also be involved that warranted further studies.

## Conclusion

In conclusion, despite the above-mentioned caveats, our findings contribute to the existing literature in the following respects. First, nursing educators should be aware of the existence of distinct empathy profiles among nursing students. In empathy and attitude interventions, top priority should be given to nursing students with low empathy. Second, educational practitioners should pay equal attention to both knowledge-providing and empathy-building trainings to reduce negative attitude toward older people, with the ultimate purpose of fostering age-friendly healthcare and high-quality caring without ageism.

## Data Availability Statement

The raw data supporting the conclusions of this article will be made available by the authors, without undue reservation.

## Ethics Statement

The studies involving human participants were reviewed and approved by the Institutional Review Boards and Ethics Committee of Xinxiang Medical University. The patients/participants provided their written informed consent to participate in this study.

## Author Contributions

G-YY, Y-YL, Z-MZ, BZ, and MG made a substantial contribution to the concept or design of the work, or acquisition, analysis, or interpretation of the data, drafted the article or revised it critically for important intellectual content, approved the version to be published, and have participated sufficiently in the work to take public responsibility for appropriate portions of the content. All authors contributed to the article and approved the submitted version.

## Conflict of Interest

The authors declare that the research was conducted in the absence of any commercial or financial relationships that could be construed as a potential conflict of interest.

## Publisher’s Note

All claims expressed in this article are solely those of the authors and do not necessarily represent those of their affiliated organizations, or those of the publisher, the editors and the reviewers. Any product that may be evaluated in this article, or claim that may be made by its manufacturer, is not guaranteed or endorsed by the publisher.
