# Supplementary material for: The Moderating Role of Empathy Profiles in the Relationship Between Knowledge About Aging and Attitudes Toward Older Adults Among Nursing Students
Source: Front Psychol. 2021 Oct 18;12:713271. doi: 10.3389/fpsyg.2021.713271 (PMC8558622; doi:10.3389/fpsyg.2021.713271)
Supplement: Supplementary file 1 [file Table_1.DOCX]

Supplementary Material

**Supplementary Table 1**

Comparisons of related factors and KAOP among nursing students (*n* = 622).

| Characteristics | | *M* | SD | *t/F* | *p* |
| --- | --- | --- | --- | --- | --- |
| Gender | Male (142) | 163.091 | 19.643 | -1.391 | 0.165 |
|  | Female (480) | 165.525 | 17.895 |  |  |
| Residence | Rural (441) | 163.655 | 18.206 | -2.808 | 0.005 |
|  | Urban (181) | 168.171 | 18.255 |  |  |
| Only child | Yes (97) | 169.897 | 17.512 | 2.900 | 0.004 |
|  | No (525) | 164.059 | 18.338 |  |  |
| Having taken geriatrics-related courses | | | | | |
|  | Yes (333) | 165.213 | 17.324 | 0.353 | 0.724 |
|  | No (289) | 164.689 | 19.433 |  |  |
| Raised by grandparents in childhood | | | | | |
|  | Yes (161) | 168.018 | 16.928 | 2.463 | 0.014 |
|  | No (461) | 163.905 | 18.683 |  |  |
| Experiences of taking care of older adults | | | | | |
|  | Yes (162) | 166.907 | 17.491 | 1.567 | 0.118 |
|  | No (460) | 164.287 | 18.574 |  |  |
| Living with older adults such as grandparents | | | | | |
|  | Yes (248) | 165.137 | 18.924 | 0.186 | 0.853 |
|  | No (374) | 164.858 | 17.935 |  |  |
| Having good relationships with older adults such as grandparents | | | | | |
| 1 | Good (508) | 167.041 | 17.729 | 21.357 | < 0.001 |
| 2 | Normal (107) | 156.682 | 17.774 | 1 vs. 2 | < 0.001 |
| 3 | Bad (7) | 141.286 | 18.874 | 1 vs. 3 | < 0.001 |
|  |  |  |  | 2 vs. 3 | 0.027 |
| Contacting with older adults such as grandparents | | | | | |
| 1 | Frequently (322) | 168.721 | 18.064 | 15.983 | < 0.001 |
| 3 | Normal (208) | 162.058 | 17.733 | 1 vs. 2 | < 0.001 |
| 3 | Non-frequently (92) | 158.424 | 17.657 | 1 vs. 3 | < 0.001 |
|  |  |  |  | 2 vs. 3 | < 0.001 |

KAOP: Total scores of Kogan's Attitude Toward Older People Scale.

**Supplementary Table 2**

Comparisons of related factors and KAOP+ among nursing students (*n* = 622).

| Characteristics | | *M* | SD | *t/F* | *p* |
| --- | --- | --- | --- | --- | --- |
| Gender | Male (142) | 84.606 | 14.043 | -0.868 | 0.386 |
|  | Female (480) | 85.581 | 11.003 |  |  |
| Residence | Rural (441) | 84.798 | 12.100 | -1.858 | 0.064 |
|  | Urban (181) | 86.724 | 10.804 |  |  |
| Only child | Yes (97) | 87.619 | 9.398 | 2.065 | 0.039 |
|  | No (525) | 84.941 | 12.110 |  |  |
| Having taken geriatrics-related courses | | | | | |
|  | Yes (333) | 86.384 | 10.465 | 2.308 | 0.021 |
|  | No (289) | 84.177 | 13.016 |  |  |
| Raised by grandparents in childhood | | | | | |
|  | Yes (161) | 87.292 | 10.389 | 2.432 | 0.015 |
|  | No (461) | 84.683 | 12.143 |  |  |
| Experiences of taking care of older adults | | | | | |
|  | Yes (162) | 86.142 | 11.517 | 0.986 | 0.325 |
|  | No (460) | 85.083 | 11.847 |  |  |
| Living with older adults such as grandparents | | | | | |
|  | Yes (248) | 84.709 | 12.885 | -1.121 | 0.263 |
|  | No (374) | 85.788 | 10.956 |  |  |
| Having good relationships with older adults such as grandparents | | | | | |
| 1 | Good (508) | 86.604 | 11.498 | 16.357 | < 0.001 |
| 2 | Normal (107) | 79.972 | 11.349 | 1 vs. 2 | < 0.001 |
| 3 | Bad (7) | 77.571 | 12.394 | 1 vs. 3 | 0.039 |
|  |  |  |  | 2 vs. 3 | 0.592 |
| Contacting with older adults such as grandparents | | | | | |
| 1 | Frequently (322) | 87.075 | 12.278 | 7.723 | < 0.001 |
| 3 | Normal (208) | 83.947 | 10.806 | 1 vs. 2 | < 0.001 |
| 3 | Non-frequently (92) | 82.544 | 11.122 | 1 vs. 3 | 0.001 |
|  |  |  |  | 2 vs. 3 | 0.003 |

KAOP: Total scores of Kogan's Attitude Toward Older People Scale. KAOP+: Positive KAOP.

**Supplementary Table 3**

Comparisons of related factors and KAOP- among nursing students (*n* = 622).

| Characteristics | | *M* | SD | *t/F* | *p* |
| --- | --- | --- | --- | --- | --- |
| Gender | Male (142) | 78.486 | 15.379 | -1.190 | 0.235 |
|  | Female (480) | 79.943 | 11.969 |  |  |
| Residence | Rural (441) | 78.486 | 15.379 | -2.295 | 0.022 |
|  | Urban (181) | 79.944 | 11.969 |  |  |
| Only child | Yes (97) | 82.278 | 11.910 | 2.236 | 0.026 |
|  | No (525) | 79.118 | 12.942 |  |  |
| Having taken geriatrics-related courses | | | | | |
|  | Yes (333) | 78.828 | 11.426 | -1.609 | 0.108 |
|  | No (289) | 80.512 | 14.242 |  |  |
| Raised by grandparents in childhood | | | | | |
|  | Yes (161) | 80.727 | 10.245 | 1.467 | 0.143 |
|  | No (461) | 79.221 | 13.604 |  |  |
| Experiences of taking care of older adults | | | | | |
|  | Yes (162) | 80.765 | 11.429 | 1.431 | 0.153 |
|  | No (460) | 79.204 | 13.275 |  |  |
| Living with older adults such as grandparents | | | | | |
|  | Yes (248) | 80.427 | 12.730 | 1.293 | 0.196 |
|  | No (374) | 79.069 | 12.882 |  |  |
| Having good relationships with older adults such as grandparents | | | | | |
| 1 | Good (508) | 80.441 | 12.549 | 9.421 | < 0.001 |
| 2 | Normal (107) | 76.710 | 13.167 | 1 vs. 2 | 0.006 |
| 3 | Bad (7) | 63.714 | 12.724 | 1 vs. 3 | 0.001 |
|  |  |  |  | 2 vs. 3 | 0.009 |
| Contacting with older adults such as grandparents | | | | | |
| 1 | Frequently (322) | 81.646 | 12.449 | 9.623 | < 0.001 |
| 3 | Normal (208) | 78.110 | 11.881 | 1 vs. 2 | 0.002 |
| 3 | Non-frequently (92) | 75.880 | 14.880 | 1 vs. 3 | < 0.001 |
|  |  |  |  | 2 vs. 3 | 0.160 |

KAOP: Total scores of Kogan's Attitude Toward Older People Scale; KAOP-: Negative KAOP.
